# An fMRI Compatible Smart Device for Measuring Palmar Grasping Actions in Newborns

**DOI:** 10.3390/s20216040

**Published:** 2020-10-23

**Authors:** Daniela Lo Presti, Sofia Dall’Orso, Silvia Muceli, Tomoki Arichi, Sara Neumane, Anna Lukens, Riccardo Sabbadini, Carlo Massaroni, Michele Arturo Caponero, Domenico Formica, Etienne Burdet, Emiliano Schena

**Affiliations:** 1 Unit of Measurements and Biomedical Instrumentation, Università Campus Bio-Medico di Roma, Via Alvaro del Portillo, 00128 Rome, Italy; d.lopresti@unicampus.it (D.L.P.); r.sabbadini@unicampus.it (R.S.); c.massaroni@unicampus.it (C.M.); 2 Division of Signal Processing and Biomedical Engineering, Department of Electrical Engineering, Chalmers University of Technology, SE-412 96 Gothenburg, Sweden; dallorso@chalmers.se (S.D.); muceli@chalmers.se (S.M.); 3 Centre for the Developing Brain, School of Biomedical Engineering and Imaging Sciences, King’s College London, London WC2R 2LS, UK; tomoki.arichi@kcl.ac.uk (T.A.); sara.neumane@kcl.ac.uk (S.N.); 4 Paediatric Neurosciences, Evelina London Children’s Hospital, Guy’s and St Thomas’ NHS Foundation Trust, London SE1 7EH, UK; anna.lukens@gstt.nhs.uk; 5 NeuroDiderot Unit UMR1141, Université de Paris, INSERM, F-75019 Paris, France; 6 UNIACT, Université Paris-Saclay, CEA, NeuroSpin, F-91191 Gif-sur-Yvette, France; 7 Photonics Micro- and Nanostructures Laboratory, ENEA Research Center of Frascati, 00044 Frascati (RM), Italy; michele.caponero@enea.it; 8 Unit of Neurophysiology and Neuroengineering of Human-Technology Interaction (NeXt Lab), Università Campus Bio-Medico di Roma, Via Alvaro del Portillo, 00128 Rome, Italy; d.formica@unicampus.it; 9 Department of Bioengineering, Imperial College London, London SW7 2AZ, UK; e.burdet@imperial.ac.uk

**Keywords:** fiber Bragg grating sensors (FBGs), functional magnetic resonance imaging (fMRI), grasping actions detection, motor assessment, MR-compatible measuring systems

## Abstract

Grasping is one of the first dominant motor behaviors that enable interaction of a newborn infant with its surroundings. Although atypical grasping patterns are considered predictive of neuromotor disorders and injuries, their clinical assessment suffers from examiner subjectivity, and the neuropathophysiology is poorly understood. Therefore, the combination of technology with functional magnetic resonance imaging (fMRI) may help to precisely map the brain activity associated with grasping and thus provide important insights into how functional outcomes can be improved following cerebral injury. This work introduces an MR-compatible device (i.e., smart graspable device (SGD)) for detecting grasping actions in newborn infants. Electromagnetic interference immunity (EMI) is achieved using a fiber Bragg grating sensor. Its biocompatibility and absence of electrical signals propagating through the fiber make the safety profile of the SGD particularly favorable for use with fragile infants. Firstly, the SGD design, fabrication, and metrological characterization are described, followed by preliminary assessments on a preterm newborn infant and an adult during an fMRI experiment. The results demonstrate that the combination of the SGD and fMRI can safely and precisely identify the brain activity associated with grasping behavior, which may enable early diagnosis of motor impairment and help guide tailored rehabilitation programs.

## 1. Introduction

Human infants exhibit a range of spontaneous movements and primitive reflexes as they develop across the first few months following birth. Among these, the grasping reflex is considered a key behavior that enables their first interactions with their surroundings [1]. The absence of such a reflex during the first days after birth or its long-lasting persistence after sixth months of age is associated with underlying brain injury and considered to be predictive of later neuromotor impairment, e.g., cerebral palsy (CP) [2]. For this reason, clinical assessment tools for neurodevelopment disorders and brain abnormalities in the infant period (particularly for those born preterm or at high risk of developing later CP) usually incorporate a component that involves eliciting the grasp reflex [3,4].

Clinical assessment of palmar grasp is currently performed by applying light pressure on the infant’s palm with an object or the examiner’s finger to induce hand closure. This approach is entirely qualitative and based on the use of functional scales and the examiner’s expertise [5] and is therefore potentially affected by inter-rater variability [6]. Furthermore, whilst clinicians and physiotherapists may develop discriminative experience about the quality of this reflex, they will still lack the sensitivity to identify subtle features not perceivable by a human observer (e.g., the discrimination of active/passive touch or the quantitative measurement of grasping strength and holding time). Therefore, a quantitative measure of grasping actions may provide new and valuable information about infant motor behavior. Consequently, there is a pressing need for new technologies that could enable the accurate measurement of grasping behavior and thus objectively evaluate sensorimotor function in the newborn. The use of such a technology could provide further insights into the relationship between motor behavior and underlying patterns of brain activity when combined with neuroimaging techniques [7]. This knowledge may be crucial in the context of early brain injury at a time when there is a high capacity for compensatory neural plasticity and thus the potential to improve functional outcome through patient-specific tailored therapies [8,9].

Functional magnetic resonance imaging (fMRI) allows for the non-invasive study of human brain function and has been successfully applied even with very young subjects [10,11,12,13]. In a typical fMRI experiment, brain responses when performing a task (which can be either an active action or passive stimulation) are identified by measuring temporal changes in the blood oxygen level dependent (BOLD) signal. However, a large challenge in performing fMRI studies with newborn infants is designing and effectively providing a task to this inherently uncooperative population. Technology can potentially resolve this difficulty and enable fMRI studies by allowing precise patterns of safe stimulation or accurate measures of spontaneous behavior while the subject is inside the scanner [6,8].

With the advance of sensor technology, several solutions have been proposed to quantitatively assess grasping behavior in infants and thus remove subjectivity. The majority of these studies have utilized technologies to assess grasping behavior in terms of strength and holding time, as well as to investigate the relationship between grasping pattern and intrinsic (e.g., infant gender, weight, premature births) and extrinsic factors (e.g., object shape and texture) [14,15]. The aforementioned measuring systems have used pressure transducers based on electrical components which are unusable inside the MRI scanner [6,16,17,18,19]. MR-compatible technology requires unconventional solutions to ensure safety due to the strong static magnetic field and avoid electromagnetic interference (EMI), with further attention required for successfully imaging infant subjects. Fiber Bragg gratings (FBGs) may be a solution to overcome this issue [20]. They are highly sensitive, small-sized, light, non-toxic, and immune to EMI [21]. Therefore, these features meet the technical and clinical requirements (e.g., MR-compatibility and safety) of devices for detecting and measuring grasping in preterm and term neonates both inside and outside the MRI scanner [6].

To date, only a few studies have systematically investigated functional brain activity in newborn infants using assistive technologies [8,9]. In [16], a customized robotic interface was used to passively move the limbs of preterm infants and identify the corresponding brain responses within the sensorimotor cortex. This study showed that the human brain is already functionally organized even at a very young age and paved the path for future investigations of task-related functional responses in preterm infants. Whilst the authors investigated the somatosensory response related to passive movements, they were unable to detect grasping actions.

The present study aims to develop an FBG-based measuring system (hereinafter called the smart graspable device (SGD)) to detect grasping actions in newborns. The SGD consists of an FBG sensor encapsulated into a soft silicone matrix. The FBG ensures that the device has EMI and the ideal safety profile for working with infants and inside the MRI environment. The device softness and cylindrical shape mimicking the ones of a finger additionally provides easy affordance and usability. Assessing grasp with the proposed device is based around the principle of the transduction of external forces (F_ext_) applied by an infant’s hand on the device into grating strain (ε) via squeezing and releasing of the silicone. In this paper we firstly described the design and fabrication of the proposed device so as to fulfill all technical and clinical requirements according to the target population (i.e., preterm and term newborns). Then, the sensitivity of the SGD to F_ext_ was estimated. Finally, two preliminary trials were carried out to investigate the performances of the proposed device when used by a preterm infant in the neonatal intensive care unit and an adult during an fMRI experiment.

## 2. Basic Requirements and Components of the Smart Graspable Device

### 2.1. Technological and Clinical Requirements

Designing a sensing device able to detect natural grasping actions and work simultaneously within an fMRI experiment is very challenging. Furthermore, there are additional constraints set by the target population (i.e., preterm and term newborn infants), which are not fulfilled by the majority of devices currently proposed in the literature [6,22]. In addition, the electromagnetic field inside the MRI environment can affect the working capability of most electronic devices proposed for grasp measurements and can induce currents in metal loops leading to infant contact burns [23,24]. In the same way, ferromagnetic elements widely used as components of electronic graspable devices may cause artefacts on the MRI images themselves, affecting diagnostic image quality [25].

As newborn infants are inherently uncooperative subjects, a further requirement of the device is that their natural movements can be safely measured under natural conditions [6,19]. To allow for handling by a newborn infant, the proposed tool should be small and lightweight, and its shape should be appropriate to improve engagement and encourage palmar grasping [26]. Moreover, high sensitivity to a low range of loads is necessary to detect the hand closure of a newborn infant, as well as being biocompatible and easily cleaned to reduce the risk of cross-infection between subjects [15,27].

To meet all of these technical and clinical requirements, an FBG sensor was chosen as the sensing element. A flexible and non-toxic silicone rubber (i.e., Dragon Skin™ 10) was used as a squeezable matrix to encapsulate the FBG sensor [28]. Lastly, a polylactic acid (PLA) structure characterized by a linkage mechanism filled with the soft silicone was designed according to the index finger dimensions of an adult human, as this is typically used to insert into the infant’s palm to elicit the grasping reflex. The proposed solution is highly sensitive, robust, safe, affordable, and infant-friendly.

### 2.2. Fiber Bragg Gratings Working Principles

An FBG sensor is a distributed Bragg grating inscribed into a short segment of an optical fiber produced by creating a perturbation of the effective refractive index (η_eff_) of the fiber core. In its simplest form, this periodic perturbation is sinusoidal with Λ, the constant grating pitch.

Generally, an FBG works in reflection as a notch filter; when a broadband spectrum of light is guided within the core and hits on the grating segment, a smooth Gaussian-shaped narrow spectrum is reflected and represents the output of the FBG. The center of the reflected Gaussian peak is known as the Bragg wavelength (λ_B_) and satisfies the Bragg condition [29].

λ_B_ = 2 η_eff_Λ (1)

Strain along the fiber longitudinal axis (ε) and temperature changes (ΔT) induce variations of Λ and η_eff,_ which result in a λ_B_ shift (Δλ_B_) as in

Δλ_B_ = λ_B_ [(1 − P_e_) ε + (α_Λ_ + ξ) ΔT] (2)

The first term of Equation (2) represents the ε effect on the grating, with Pe the effective strain-optic constant; the second term represents the ΔT effect, with α_Λ_ and ξ denoting the thermal expansion and the thermo-optic coefficients of the fiber, respectively. When the effects of ΔT are negligible, Equation (2) in [29] can be rewritten as

Δλ_B_ = λ_B_ (1 − P_e_) ε (3)

In this work, the SGD is able to detect grasping forces applied by a newborn infant through the compression of the silicone encapsulation that allows transducing loads applied on the device into longitudinal ε experienced by the FBG.

Based on the need for a physical connection to a dedicated device (i.e., the FBG interrogator) for enlightening the gratings and reading their outputs, a patch cord can be used to connect the FBG-based device inside the MRI scanner to the interrogator placed in the control room. This connection allows separation of the SGD inside the MRI scanner room from the measuring circuitry located in the control room.

### 2.3. Dragon Skin™ Silicones

Dragon Skin™ materials (commercialized by Smooth On Inc., Macungie, PA, USA) are platinum care bicomponent silicone rubbers used in a variety of scenarios, including medical fields (e.g., in prosthetics as cushioning materials and in physiological monitoring for flexible sensor development [30,31]). They are highly compliant and highly flexible. Moreover, they are skin safe in compliance with ISO 10993-10 (Biological evaluation of medical devices—Part 10: Tests for irritation and skin sensitization) [28,32].

Dragon Skin™ silicones are commercialized as liquid silicone rubbers in the form of an elastomer kit containing two components (A and B). Part A contains the platinum catalyst, part B the crosslinker [33]. The manufacturer recommends mixing Dragon Skin™ silicones in the proportion of 1A:1B by weight and thinning the liquid formulation with Silicon Thinner™ to lower the viscosity of the mix for easier pouring and vacuum degassing [34]. Curing temperature and time are also defined in the technical bulletin. Dragon Skin™ silicones are commercialized in different hardnesses expressed in terms of Shore A Scale: 10 (Very Fast, Fast, Medium, Slow), 20, and 30, with curing time ranging from 4 min to 45 min according to the silicone hardness.

In this work, the mechanical properties of Dragon Skin™ 10 Medium, 20, and 30 were investigated in terms of stress–strain properties. To better quantify the compression behavior of Dragon Skin™ silicones, the Young modulus E (expressed in MPa) was calculated to facilitate the selection of the material that best satisfies all the requirements mentioned above. Considering the scenario of interest and target population, the application of low squeezing forces will induce a compression on the silicone rubber with ε values lower than 10% of the rubber sample (l_0_). Thus, the stress–strain relationship can be described by Hooke’s law [33]:

σ = E ε(4)

where σ is the stress (i.e., the external force applied to the sample per its cross-sectional area) and ε is calculated as (l − l_0_)/l_0_.

The standard ISO 7743:2017 (Rubber, vulcanized or thermoplastic—Determination of compression stress–strain properties) was used for defining the dimensions of the cylindrical pieces used for the compression tests. The test piece B (method C) with a diameter of 17.8 ± 0.2 mm and a height of 25.0 ± 0.2 mm was chosen [35]. Dragon Skin™ 10 Medium, 20, and 30 were poured into a cylindrical mold designed in Solidworks (Dassault Systemes, Waltham, MA, USA) and 3D printed using PLA. As suggested by the technical bulletin, the curing process was carried out at room temperature for 5 h, 4 h, and 16 h for Dragon Skin™ 10, 20, and 30, respectively. A total of fifteen specimens were fabricated, five pieces for each hardness level.

**Figure 1:**
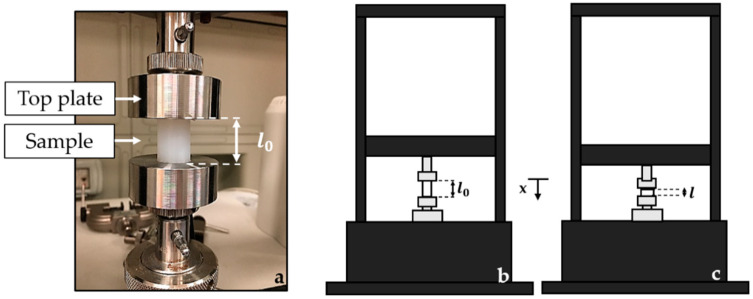
Set-up of the compression tests (**a**): the cylinder-shaped sample between the lower and the upper plates of the Instron machine and the initial sample length before (l_0_) and during the compression (l) are illustrated ((**b**,**c**), respectively). The top plate moves at constant speed parallel to the *x*-axis (**c**).

Compression tests were carried out using a testing machine (Instron^®^, Norwood, MA, USA, model 3365, load cell with a range of measurement of ±10 N, an accuracy of 0.02 N, and a resolution of 10^−5^ N) to apply controlled ε values (from 0% to 25% of l_0_ as suggested by the standard ISO 7743:2017) in a quasi-static condition (at a low displacement rate of 2 mm·min^−1^). The static assessment of each specimen was executed by positioning the cylinder-shaped sample between the lower and the upper plates of the machine, as shown in Figure 1. A total of five repetitive compression tests were carried out at room temperature for a total of twenty tests per sample. The loads and the displacements applied by the compression machine to the specimen were recorded at a sampling frequency of 100 Hz using Instron^®^ Bluehill Universal software.

The stress–strain relationships (σ vs. ε) of each Dragon Skin™ material were obtained by processing the collected data through a custom algorithm. The mean value of experimental σ (σ_exp_) and the repeatability of the system response were determined by calculating the related uncertainty across the twenty tests by considering a t-Student reference distribution with 19 degrees of freedom and a level of confidence of 95% [36]. The best fitting line of the calibration curve was obtained, and its angular coefficient was calculated to estimate E. Lastly, the linearity error was calculated by using Equation (5) in terms of the maximum linearity error (% u_L_^max^).

% u_L_^max^ = {max [σ_exp_ (ε) − σ_th_ (ε)] · σ^fs^_exp_^−1^} · 100 (5)

where σ^fs^_exp_ is the full-scale output range, σ_exp_(ε) the experimental stress experienced by the sample at a specific ε, and σ_th_(ε) is the theoretical stress obtained by the linear model at the same ε value.

**Figure 2:**
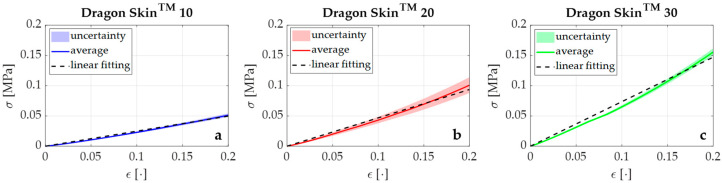
The σ–ε relationships for Dragon Skin™ 10, 20, and 30 are shown in blue (**a**), red (**b**), and green (**c**), respectively. In particular, the continuous lines represent the average σ_exp_ values, the shaded areas the related uncertainties, and the black dotted lines the σ_th_ values obtained by the best linear fitting model.

Results showed E values of 0.24 MPa, 0.47 MPa, and 0.74 MPa for Dragon Skin™ 10, 20, and 30, respectively (see Figure 2). R-square (R^2^) values higher than 0.98 were found for all the responses, and linearity errors of 5.7%, 7.8%, and 8.9% were obtained for Dragon Skin™ 10, 20, and 30, respectively.

Our results quantified the mechanical properties of Dragon Skin™ in terms of compression behavior. As expected, Dragon Skin™ 10 was found to be more flexible than Dragon Skin™ 20 and Dragon Skin™ 30. In particular, the E value of Dragon Skin™ 10 was approximately half that of Dragon Skin™ 20 (i.e., 0.24 MPa vs. 0.47 MPa) and one-third that of Dragon Skin™ 30 (i.e., 0.24 MPa vs. 0.74 MPa). The high R^2^ values (for all tests R^2^ > 0.98) indicated good agreement between the experimental data and the linear model. Moreover, the Dragon Skin™ 10 response showed the best linear behavior as testified by the % u_L_^max^ value (i.e., 5.7%), which was lower than those of Dragon Skin™ 20 (i.e., 7.8%) and Dragon Skin™ 30 (i.e., 8.9%), as shown in the respective plots in Figure 2. Finally, Dragon Skin™ 10 showed the best results in terms of uncertainty (maximum uncertainty of 0.004 MPa) when compared to Dragon Skin™ 20 (i.e., 0.01 MPa) and Dragon Skin™ 30 (i.e., 0.007 MPa).

These findings demonstrated that Dragon Skin™ 10 is best suited to meet the technical requirements of the SGD, particularly given the expected low ranges of F_ext_ applied by newborn infants, which would likely require high flexibility; Dragon Skin™ 10 allows the SGD to be easily squeezed by a newborn for the F_ext_ transduction into grating ε.

## 3. The Smart Graspable Device

### 3.1. Design and Manufacturing of the Smart Graspable Device

The idea behind the system design and manufacturing was based on the need for it to be sensitive enough to detect F_ext_ applied by the infant and able to transduce F_ext_ into grating ε measured by the FBG sensor. At the same time, the device should be robust and safe enough to be handled and grasped by a newborn infant in a variety of settings.

**Figure 3:**
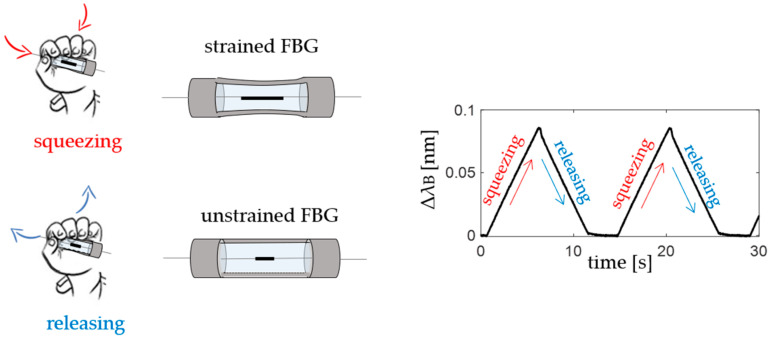
The smart graspable device (SGD) principle of working during grasping: squeezing (in red) and releasing (in blue) phases.

The medium consists of a PLA-based structure characterized by a linkage mechanism and filled by flexible silicone (i.e., Dragon Skin™ 10). The transduction mechanism exploits four-bar linkages hinged to the PLA structure ends to convert the applied F_ext_ into ε via the silicone squeezing and releasing. When the newborn infant grasps the device, the silicone is squeezed and the FBG is strained; it is then unstrained once the SGD is released again (see Figure 3).

**Figure 4:**
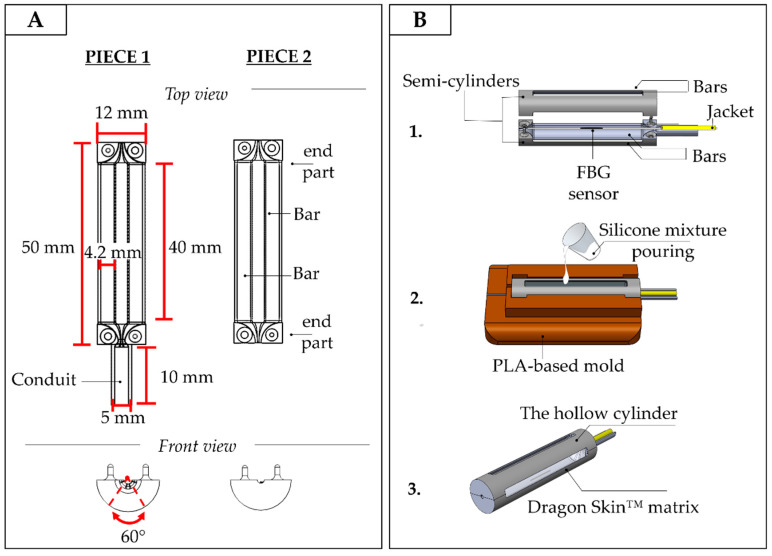
(**A**) The geometrical features of an SGD; (**B**) The main manufacturing steps: the FBG sensor positioning (step 1), the silicone mixture pouring (step 2), and the SGD removed from the mold after the rubber vulcanization (step 3).

The PLA structure is a hollow cylinder made up of two semi-cylindrical pieces (see Figure 4A,B). Each piece is constituted of two end parts perpendicularly spaced by two bar linkages (40 mm of length, 4.2 mm of width, and 1 mm of depth). The bar linkages are hinged along the ends with a uniform interval of 60° to form a symmetrical structure (Figure 4A, top and front views). One semi-cylinder has a 10-mm long conduit to accommodate the jacket (yellow cable in Figure 4B). The jacket is the last layer of protection of the optical fiber from the end of the PLA structure to the interrogation unit.

The outer diameter of the PLA structure is 12 mm, and its overall length is 50 mm, which corresponds roughly to the dimensions of a human finger. The sensing element embedded into the PLA structure consists of an FBG sensor (λ_B_ of 1547 nm and grating length of 10 mm, commercialized by AtGrating Technologies, Shenzhen, China) configured with the middle part of the optical fiber encapsulated into a Dragon Skin™ 10 silicone matrix.

The encapsulation was fabricated as follows:

1. the optical fiber was tightly suspended inside the PLA structure by firmly gluing its two ends inside the small grooves fabricated at the center of the structure ends. This configuration utilized pre-tension to keep the FBG in a stretched state in order to improve its resolution and sensitivity (stage 1 in Figure 4B);
2. the PLA structure was placed inside a mold to allow cavity filling without any silicone spilling. The silicone rubber was synthesized by mixing A and B liquid components of Dragon Skin™ polymer at a ratio of 1:1; the mix was degassed and poured into the cavity (stage 2 in Figure 4B);
3. a curing process of 5 h was carried out at room temperature, as suggested in the technical bulletin [28], to allow the silicone rubber vulcanization before extracting the SGD from the mold (stage 3 in Figure 4B).

Two nominally identical SGDs (from now on referred to as SGD^1^ and SGD^2^) were developed following the same manufacturing stages previously described.

**Figure 5:**
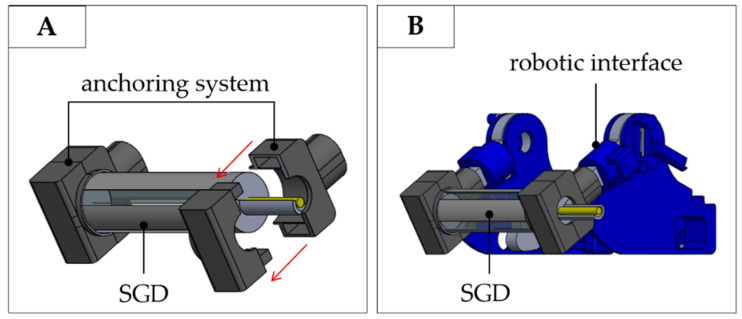
(**A**) A detail of the anchoring system elements designed to allow fitting of the SGD within an already existing robotic interface; (**B**) the SGD together with the robotic interface.

To improve the SGDs usability during fMRI, both the devices were designed to also be able to instrument an already existing fMRI-compatible robotic interface for conducting experiments with newborn infants described in [8,9]. This interface was designed to accommodate the infant’s forearm on a central non-sensitive platform and to passively guide the flexion and extension of the infant wrist while the hand was wrapped around a bar. The instrumentation of the robotic interface with the proposed SGD was performed by switching the non-sensorized handlebar with the SGD, thus allowing the investigation of brain activity related to grasping and spontaneous movements (Figure 5A,B). To fit the SGD to the robotic interface, two temporary PLA-based anchoring systems were fabricated (see Figure 5A). The anchoring mechanism was designed to allow quick and easy fitting of the SGD according to the fMRI investigation being performed.

### 3.2. Metrological Characterization of the Smart Graspable Device

**Figure 6:**
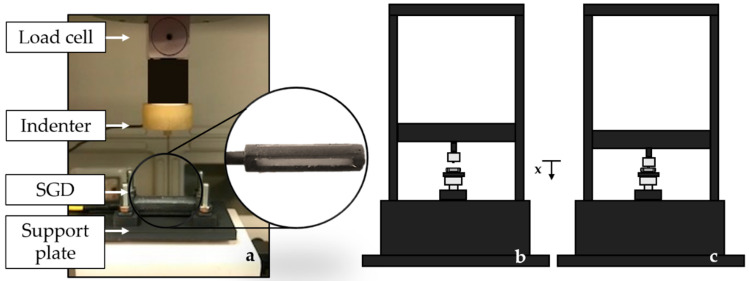
The SGD in the blocking system, the load cell, and the mechanical indenter are shown (**a**). A schematic representation of the SGD between the lower and the upper plates of the Instron machine is illustrated (**b**). The top plate moves at constant speed parallel to the *x*-axis (**c**).

To estimate SGD sensitivity to F_ext_ (S_F_), compression tests were performed by using a tensile testing machine (Instron, model 3365, load cell with a range of measurement of ±10 N, an accuracy of 0.02 N, and a resolution of 10^−5^ N). The blocking system shown in Figure 6 was designed to avoid any axial rotation of SGD during the application of loads on the SGD bars. This system consisted of a support plate with two clamps to securely lock the SGD through M4 fixings. 

Each sensor (i.e., SGD^1^ and SGD^2^) was placed on the lower base of the machine blocked to the support place by using the 3D-printed clamps. External loads in the range 0 N–2 N were applied on each bar of the SGD at a low compression rate of 2 mm·min^−1^ to simulate quasi-static conditions (see Figure 6).

The load was applied to the center of each bar by using a mechanical indenter (5 mm diameter). A total of five compression tests were performed on each of the four bars for a total of 20 tests. Once the five tests related to a single bar ended, the SGD was rotated 90° along its longitudinal axis, re-blocked to the support, and the second bar was loaded. The same procedure was repeated for the remaining two bars. The S_F_ value of each SGD was found averaging the twenty responses of all the bars to the applied loads.

During each test, the output from the tensile machine was collected at a sampling frequency of 100 Hz. The Δλ_B_ values were simultaneously recorded using the optical spectrum interrogator (si255, Hyperion Platform, Micro Optics Inc., Atlanta, GA, USA) at the same sampling frequency.

**Figure 7:**
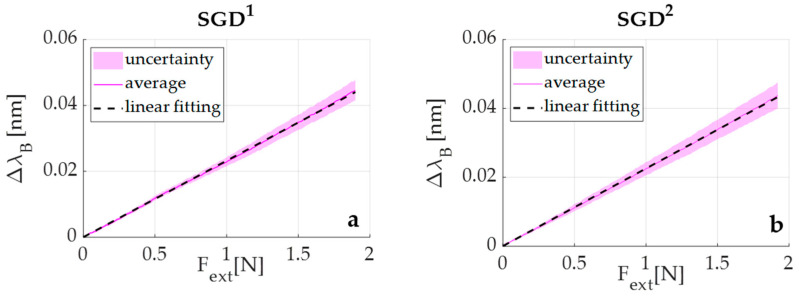
The Δλ_B_ vs. F_ext_ of SGD^1^ (**a**) and SGD^2^ (**b**): in continuous magenta lines the average Δλ_B_ vs. F_ext_ responses, in shaded magenta areas the related uncertainties, and in dotted black lines the best linear fitting.

The calibration curve (Δλ_B_ vs. F_ext_) was obtained by processing the collected data through a custom algorithm to evaluate the average value of Δλ_B_ vs. F_ext_. The first step was the synchronization of the experimental values of Δλ_B_ and F_ext_. Then, both the average value and the expanded uncertainty of Δλ_B_ were calculated across the twenty tests. The expanded uncertainty was calculated by using a t-Student reference distribution (with 19 degrees of freedom and a level of confidence of 95%). Finally, a linear regression to find the equation of the line which best fits the experimental data (i.e., Δλ_B_ vs. F_ext_) was performed. Its slope represents the S_F_ value for SGD^1^. The same procedure was followed for SGD^2^ (see Figure 7).

Results showed a S_F_ value of ~0.23 nm·N^−1^ for both devices, suggesting a good reproducibility of the fabrication process. Moreover, an R^2^ > 0.99 indicated excellent agreement between the experimental data and the linear fitting model.

## 4. Experimental Validation of the Smart Graspable Device

To assess the proposed SGDs performance in a real scenario, two explorative tests were performed with a newborn infant and an adult subject. Given the high agreement in S_F_ values between SGD^1^ and SGD^2^, SGD^1^ was used for the following tests and for simplicity is now referred to as SGD. First, an explorative test was performed on a healthy newborn infant in the neonatal intensive care unit of St. Thomas Hospital (London, UK) to test compatibility of the SGD and assess its ability to detect a newborn infant’s grasp. A further explorative trial within an fMRI experiment was then carried out to validate the device performances inside the MRI scanner and to confirm that activation within the brain areas associated with the grasping action detected by SGD could be identified with fMRI.

### 4.1. Experimental Trial on a Newborn: Protocol and Results

To assess the capability of SGD to detect grasping behavior in newborn infants, three healthy infants were recruited at St Thomas’ Hospital (London, UK). Of them, only one infant (gestational age at birth: 36 weeks + 6 days, postmenstrual age at the time of recording: 37 weeks + 2 days) was in a suitable awake state at the time of the recording. The study was approved by the NHS research ethics committee (REC code: 12/LO/1247), and informed written consent was obtained from parents prior to participation. A neonatal physiotherapist under the supervision of a physician handled the SGD and applied light pressure on the infant’s palm to induce hand closure around the device and elicit grasping behavior. Data from the SGD were collected using the FBG interrogator (si425, Micron Optics Inc., Atlanta, GA, USA) at a sampling frequency of 250 Hz, and a video used as reference was simultaneously recorded using a camera (Handycam, Sony, Minato-ku, Tokyo, Japan).

**Figure 8:**
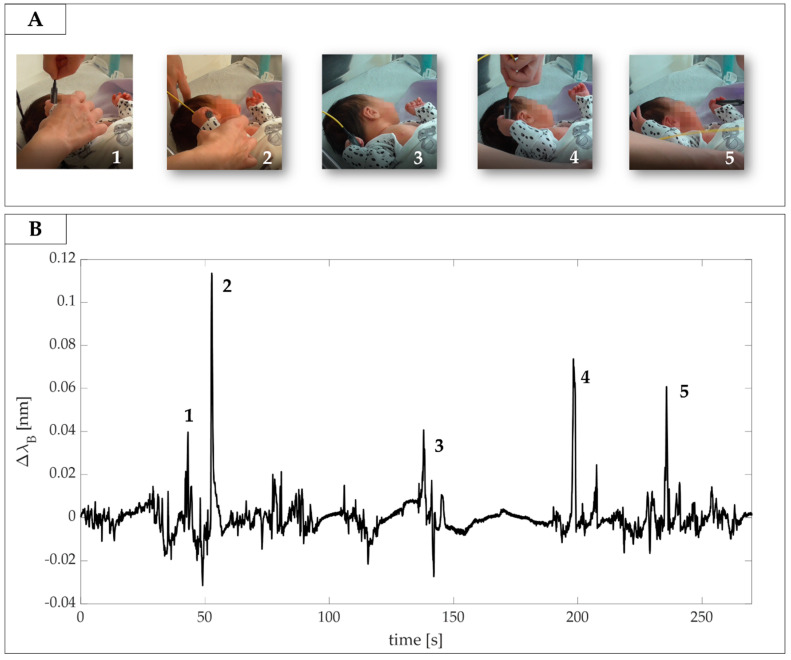
(**A**) Video frames of grasping actions identified by the clinicians; (**B**) the related signals collected by the SGD. Grasps actions are enumerated from 1 to 5.

After data acquisition, the physiotherapist and the physician checked the recorded video to identify the time windows corresponding to the grasping action performed by the newborn infant. A total of five grasping events were identified (see Figure 8A).

Those time windows were then used to highlight changes in the SGD output associated with grasping actions. The signal showed evident peaks during the specific time windows related to presumed SGD squeezing and releasing actions (see Figure 8B). Results showed Δλ_B_ values ranging from 0.04 nm to 0.11 nm that corresponded to forces ranging from ~0.17 N to ~0.48 N.

### 4.2. Experimental Trial on an Adult during fMRI: Protocol and Results

The second trial was performed on a healthy volunteer (32 years old, adult female volunteer) inside a 3 Tesla MRI scanner (Philips Achieva, Best, The Netherlands) located at St Thomas Hospital with a 32-channel receive head coil. High resolution structural T1-weighted and T2-weighted images were acquired for image registration purposes. BOLD contrast fMRI data with an EPI GRE sequence with parameters: x/y/z resolution: 3.5 mm × 3.5 mm × 6 mm; TR: 1500 ms; TE: 45 ms; FA 90°.

The subject was studied with her right hand fitted inside the instrumented robotic interface described in [8,9] with the right index and middle fingers strapped to the SGD on the handle bar.

**Figure 9:**
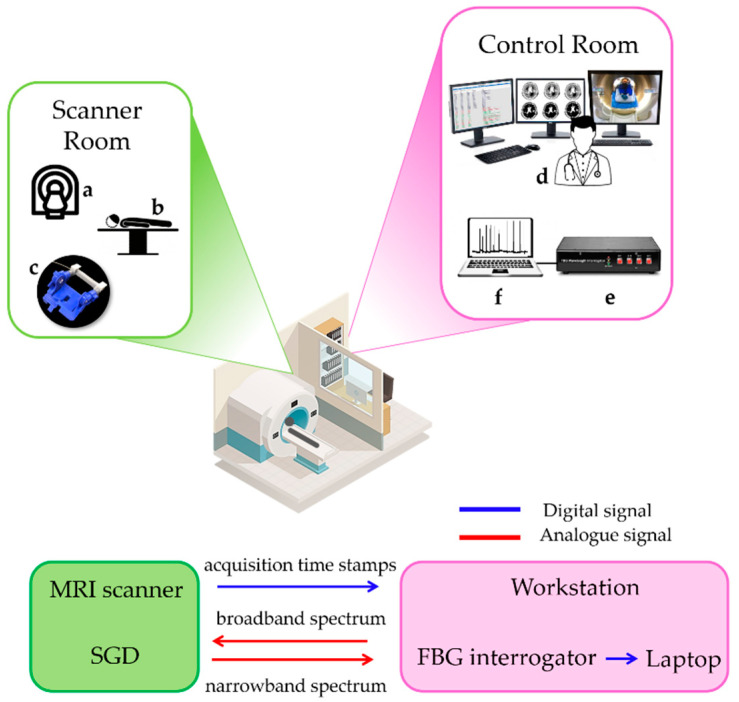
Experimental set-up of fMRI trial with the data flow: the MRI scanner (**a**), the patient (**b**), and the robotic interface instrumented by the SGD (**c**) in the scanner room, and the workstation (**d**), the FBG interrogator (**e**), and the laptop (**f**) in the control room. Digital and analogue data flows between the scanner and control rooms are shown using blue and red arrows, respectively.

The subject was then asked to use their two fingers to apply a brief force on the SGD at spontaneous and random times during an acquisition session lasting 225 s (corresponding to 150 images). The SGD signal was collected using the FBG interrogator (si425, Micron Optics Inc., Atlanta, GA, USA) at a sampling frequency of 250 Hz, and the recording started synchronously with the fMRI image acquisition so that the timing of task could be related to the fMRI timeseries. The task was designed to simulate a situation in which the experimenter is unaware of the timing of the task inside the scanner, as would be the case when studying spontaneous motor behavior in infants but can obtain this information from the output of the SGD. The experimental set-up and data flow are shown in Figure 9.

**Figure 10:**
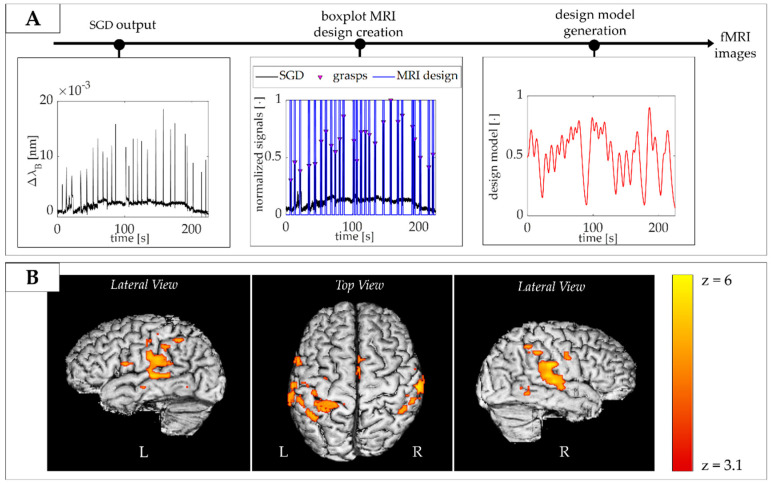
(**A**) Flow of data analysis. The peak in Δλ_B_ coming from the SGD were used to infer the timing of the action and create a boxplot MRI design. This was convolved with the hemodynamic response function (HRF) to generate the design model for the general linear model (GLM); (**B**) Significant cluster of functional response to the task (in red and yellow) overlaid onto the subject’s T1-weighted brain image with the z-score map.

The acquisition volume trigger markers (via a TTL pulse) were transmitted to the scanner workstation and used offline to synchronize the fMRI data with the signal recorded by the SGD. Data from the SGD were analyzed in the MATLAB R2019b environment (Mathworks, Natick, MA, USA). As shown in Figure 10A, the device was able to detect the force applied by the subject without prior knowledge of its timing (i.e., 26 actions in the SGD output across the period of acquisition) from which the event-related occurrence of the task could be defined for the fMRI data analysis.

In order to identify which voxels of the brain images were active during the task, it is possible to use a simple general linear model (GLM) to fit the BOLD time series within each voxel with a temporal model of the predicted activation, and the strength of each fit is used to generate a z-statistics map across the whole brain (z-score map in Figure 10). The predicted activation used as a model is built as the convolution of the experimental design (a vector that represents the timing of action vs. rest) and the hemodynamic response function (HRF) that act as a temporal smoothing kernel. The SGD-derived task pattern was then expressed in a binary vector form (with 1’s representing action and 0’s representing rest) where the events were identified using the *findpeaks* function on the normalized SGD signal in the MATLAB environment (see Figure 10A). Each event was represented by 8 ms centered at the peak of the action, and the binary vector was then convolved with the canonical hemodynamic response function (HRF) to generate the design model for the general linear model fMRI analysis (see Figure 10A). MRI data were processed using tools implemented in the FMRIB software library (FSL) in [37] following a standard pipeline which included high pass temporal filtering (with 0.02 Hz cut-off frequency), MCFLIRT rigid body motion correction, slice timing correction, brain extraction using BET, spatial smoothing (Gaussian of FWHM 5 mm), univariate general linear model (GLM) with additional 6 motion parameters derived from the rigid body motion correction as confound regressors, and cluster correction (p threshold 0.05). As predicted, significant clusters of positive BOLD activity were located in the primary contralateral (left) somatosensory and motor cortices and supplementary motor area correlating to the task performed by the subject, and the main cluster of activation was located at the hand knob on the precentral gyrus (see left (L) and right (R) lateral and top view on the 3D rendered brain images in Figure 10B).

## 5. Discussion

The key elements of the present study are the development of a novel smart device (i.e., the SGD), which can quantitatively measure the grasping behavior of newborn infants even within the MRI scanner environment. The design of the proposed device was guided by technical and clinical requirements predicated by the target population and with the intended use including small size, lightweight, high sensitivity, biocompatibility, and safety. In addition, the SGD was also designed to easily instrument an existing robotic interface used for fMRI studies of newborn infants.

To the best of our knowledge, the proposed SGD is the first fMRI-compatible device based on FBG able to detect and measure palmar grasp in preterm and term infants. The SGD is shaped and sized appropriately to be handled by a newborn infant’s hand, easily squeezed, and able to detect low grasping F_ext_. Furthermore, the FBGs’ EMI compatibility ensures safety and working capability both outside and inside the MRI environment.

Two identical devices were fabricated (i.e., SGD^1^ and SGD^2^). Both the responses of SGD^1^ and SGD^2^ to F_ext_ showed a linear behavior, which reached an average S_F_ value of ~0.23 nm·N^−1^. This finding confirmed the high reproducibility of the manufacturing process as well as the capability of the SGD to detect the low ranges of F_ext_ applied by the target infant population. Two pilot tests were carried out to investigate the performance of the proposed SGD on a newborn infant in the neonatal intensive care unit and on an adult during an fMRI experiment. These preliminary validation experiments revealed that the SGD was capable of working in both cases, namely on a non-collaborative subject (i.e., the newborn infant) and within the MRI environment.

In the literature, pioneering work describing grasping behavior in infants began in the 1930s [38,39,40]. These first studies were based on functional scales or on the direct observation of infant responses to the application of light pressure on the palm [41]. These methods suffer from several limitations, including examiner subjectivity and incapability of quantifying grasping actions in terms of strength and duration. With the advancement of technological devices, novel methods emerged for assessing motor function in early infancy [7,14,15,16,18,19,42,43]. In particular, grasping behavior was studied in infants in terms of strength and holding time, with some studies proposing novel systems to investigate the relationship of these variables with intrinsic (e.g., infant sex [15], weight [27], preterm birth [1]) and extrinsic factors (e.g., object shape and texture [7]). Moreover, systems for objective assessment of grasping actions were developed to detect early abnormal neuromotor development and based on the premise that this could guide prompt intervention to improve functional outcome [43,44]. The main findings of the state-of-the-art showed a higher grasping strength and pronounced handedness symmetry in males more than females [15]; a decrease in holding time when the same object was repetitively put in the newborn infant’s hand and an increase when its shape and smoothness changed [7]; and longer holding times in preterm in comparison to term neonates with significant differences related to sex [15].

Existing technological tools developed for measuring grasping in newborn infants can be grouped into devices worn on the examiner’s finger [18] or those that are directly handled by an infant [16,18,19,43]. Very few of these systems were also specifically designed to be used by newborn infants in the first days following birth. All of these systems are based on pressure transducers with electrical components (i.e., piezoresistive sensors [16], force sensing resistors (FSR) [19], capacitive sensors [18], and conductive polymer layers [43]). These features do not allow their employment inside the MRI scanner. In contrast, our device is designed to be directly handled by a newborn infant and can be used in fMRI owing to the FBG sensor EMI immunity and the SGD’s affordable shape.

In [16], as in our study, the device was designed to be directly grasped by a newborn infant without active involvement from the examiner during the experimental trial. An electrical sensing element (i.e., a piezoresistive pressure transducer) was used to develop a ring-shaped device consisting of a silicone-filled chamber hung within a rigid case and connected to the transducer (total diameter of 93 mm). This allowed for the measuring of infant grasping activity generated by chamber internal pressure changes due to the silicon squeezing. Our device is different, as the proposed SGD is based on optical technology instead of electrical components, is softer (10 A vs. 50 A), and is considerably lighter (8 g vs. 115 g). All of these features make our system more suitable for studying a wider range of infant populations including preterm and term infants.

Future studies will aim to investigate the feasibility of SGD assessment on a wider range of newborn infants both inside and outside the MRI scanner. Moreover, two SGDs can be used together in longitudinal studies to study the emergence of hand dominance and (bi)manual grasping to investigate developmental motor disorders resulting from localized brain injury. Further investigation may also be focused on optimizing the SGD for use with older infants, for instance across their first year as the transition to volitional prehension occurs. These studies will require a remodulation of system requirements (e.g., change in dimensions, FBG numbers, and silicone hardness) to optimize the SGD measuring properties for a different target population. Lastly, changes in grasping variables according to age, sex, and object texture could be performed to provide new insights into behavioral neurophysiology and neuropathology.

## 6. Conclusions

This paper described the first fMRI-compatible technological solution (SGD) based on FBG for detecting grasping actions in newborn infants. The use of such a device in conjunction with fMRI can shed new light on associated cerebral processes and may provide novel insight into the neuromotor impairments that result from neonatal brain injury. This knowledge may be crucial in the context of prompt diagnoses of atypical task-related brain activations and early brain injuries such as CP, at a time when there is a high capacity for compensatory neural plasticity. Furthermore, the technology-aided assessment of neurodevelopmental deviation from normal physiological development can provide useful biomarkers for establishing patient-tailored therapies and optimizing the rehabilitation outcomes.
